# Parental Perceptions and Family Impact on Adolescents’ Oral Health-Related Quality of Life in Relation to the Severity of Malocclusion and Caries Status

**DOI:** 10.3390/children12040425

**Published:** 2025-03-28

**Authors:** Berfin Karbeyazgün Çınar, Rosaria Bucci, Vincenzo D’Antò, Simona Cascella, Roberto Rongo, Rosa Valletta

**Affiliations:** School of Orthodontics, Department of Neurosciences, Reproductive Sciences and Oral Sciences, University of Naples Federico II, Via Pansini, 5, 80131 Naples, Italy; berfinkarbeyazgun.cinar@unina.it (B.K.Ç.); vincenzo.danto@unina.it (V.D.); si.cascella@studenti.unina.it (S.C.); roberto.rongo@unina.it (R.R.); valletta@unina.it (R.V.)

**Keywords:** quality of life, oral health, parental perception, family impact, adolescents, malocclusion severity, caries, cultural adaptation

## Abstract

Background/Objectives: Parents/caregivers’ reports are valuable because they frequently play a crucial role in making decisions concerning a child’s health, and their perspectives can significantly impact treatment choices. Furthermore, negative effects of oral health issues extend beyond just the adolescent patient, having an impact on family life. The aim of this study is to explore the perception of parents/caregivers regarding their children’s oral-health-related quality of life (OHRQoL) and how the OHRQoL of adolescents impacts their family life. Methods: The Parental-Caregiver Perception Questionnaire-16 (P-CPQ-16) and the Family Impact Scale-8 (FIS-8) were administered to 160 parents/caregivers of adolescents aged 10–18 years old at the Dental Clinic of the University of Naples Federico II (Italy). Adolescents’ oral health status was recorded using the Decayed Missing and Filled Teeth (DMFT/dmft) index and Dental Aesthetic Index (DAI). Results: FIS-8 and P-CPQ-16 scores showed no significant differences across DAI and DMFT/dmft subgroups. However, regression analysis found a significant association between social well-being and total P-CPQ-16 scores with the DAI and DMFT index. Spearman’s correlation showed statistical significance only for the social well-being domain of P-CPQ-16 with respect to DAI scores. Conclusions: Parents/caregivers perceived a difference in their children’s OHRQoL according to different severities of malocclusion and dental caries; however, they did not report any impact on the family’s quality of life. Since adolescents often visit dentists due to parental influence, involving parents who perceive a lower quality of life in children with severe malocclusions or compromised oral health is crucial for effective care.

## 1. Introduction

Research on oral-health-related quality of life (OHRQoL) examines the impact of oral health outcomes on oral symptoms, functional impairments, emotional well-being, and social interactions [1]. Researchers have indicated that the assessment of OHRQoL can play a significant role in evaluating treatment needs, prioritizing care, and assessing the outcomes of treatments [2].

In recent years, there has been increasing interest in the assessment of OHRQoL in children and adolescents, as oral problems can have a detrimental effect on young people and their families [3,4]. This is particularly relevant during adolescence, a time when social relations often shift from family to friends, and physical appearance and self-image are important considerations [5]. The challenges in assessing OHRQoL in children, arising from numerous changes in this period of life, could be mitigated by utilizing a proxy, such as a parent or caregiver, to provide reports on the child’s quality of life [6]. Beyond serving as an assisting tool, acquiring parents’ reports are valuable because parents frequently play a crucial role in making decisions concerning a child’s health, and their perspectives can significantly impact treatment choices [7]. Moreover, healthcare often caters to the needs of parents rather than those of children.

Furthermore, the negative effects of severe oral health issues extend beyond just the adolescent patient. The involvement of parents is essential for the health and well-being of children. Hence, it is important to assess the impact on family life regarding the OHRQoL of children. Parents/caregivers may feel guilty about their child’s condition, and there may be conflicts within the family and missed work due to the child’s oral health problems; these issues should not be ignored, so including parents into OHRQoL studies could lead to more reliable outcomes [8]. This highlights how important it is to confirm family effects, in addition to parental views and self-reported measures of children’s quality of life [9].

The Parental-Caregiver Perceptions Questionnaire (P-CPQ) [10] and Family Impact Scale (FIS) [11] are child OHRQoL tools [12] that are filled out by parents/caregivers. The P-CPQ measures the viewpoint of the parent or caregiver [10] about their child’s OHRQoL, while the FIS measures how a child’s oral health affects the family’s quality of life [11]. Shorter versions of the questionnaires were found to have good psychometric value and were suggested to be preferred over the original long versions due to less burden caused to the respondents [13]. Healthcare professionals and researchers are more likely to utilize them frequently, and the likelihood of respondents providing incomplete data is reduced.

The aim of the present study was to measure parental/caregivers’ perceptions of the impact of children’s OHRQoL, based on the severity of children’s malocclusion in the aesthetic region and the dental caries status of their sons/daughters, in addition to measuring the impact of children’s orofacial conditions on the family. The null hypothesis was that there was no difference in the parental perceptions of a child’s OHRQoL according to the different degrees of malocclusion severity and dental caries status.

## 2. Materials and Methods

**Figure 1:**
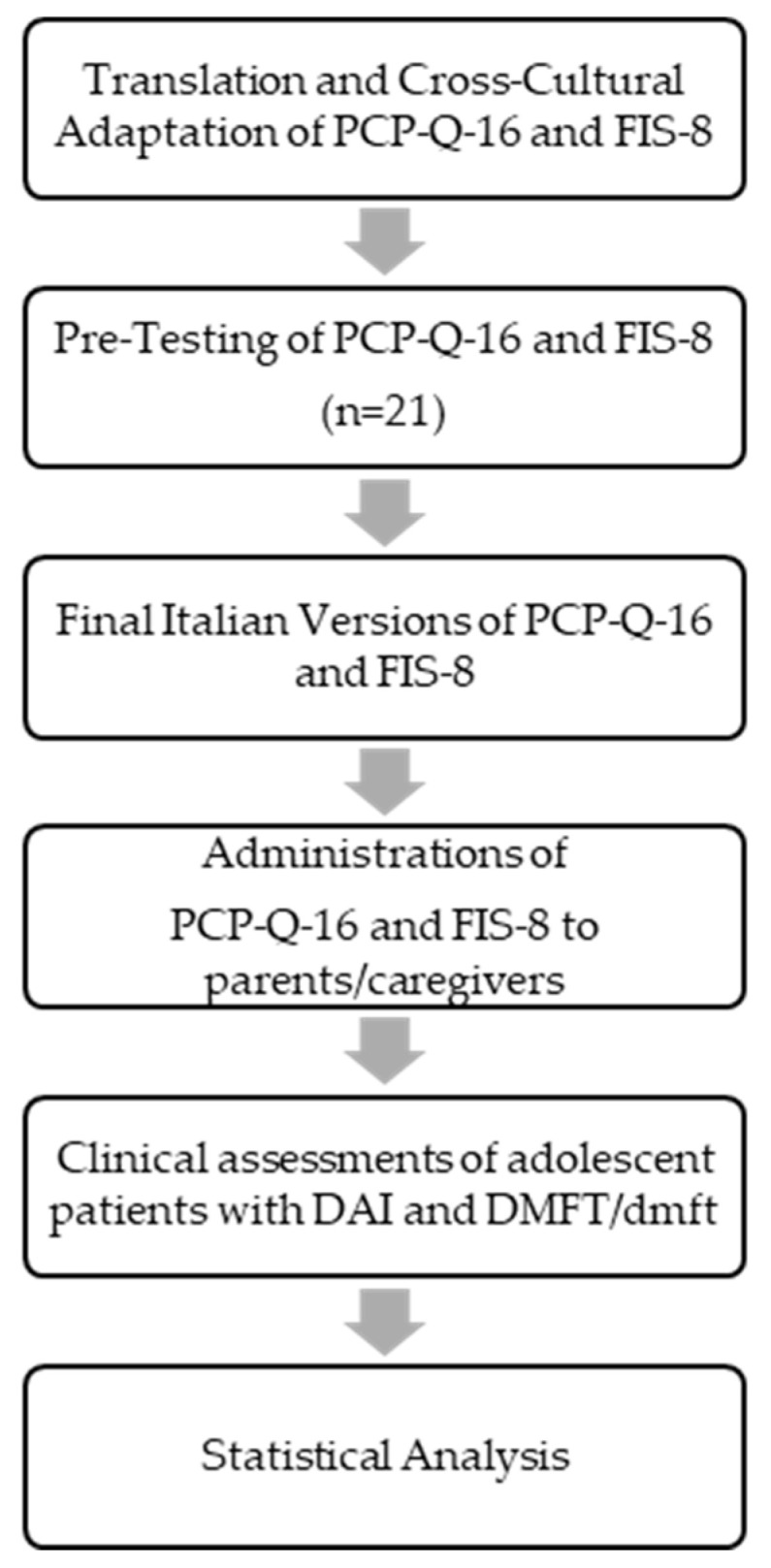
Flow chart of study design.

This study was conducted after obtaining ethical approval with data collected at the section of Orthodontics of University Naples Federico II (ethical approval number 146/2023). Written informed consent was obtained from all participants before enrollment. The study design is summarized in Figure 1.

Adolescents presenting at the Orthodontic Clinic of the University of Naples Federico II (Naples, Italy) for a first consultation between April 2023 and August 2023 were enrolled, along with their parents/caregivers.

Inclusion criteria required participants to be in good general health, to be aged 10–18 years, whose parents/caregivers had sufficient proficiency in Italian to complete the questionnaires, and who accepted to be enrolled in the study.

Exclusion criteria were as follows: patients with genetic disorders, intellectual and/or physical disabilities that prevented questionnaire completion, current or past orthodontic treatment, and a history of severe oral surgery.

The Parental-Caregiver Perceptions Questionnaire (P-CPQ-16) and Family Impact Scale (FIS-8) were completed by parents/caregivers in a self-administered manner while waiting for their child’s dental examination. A digital version of the questionnaires, including socio-demographic information, was provided using a QR code. To ensure clarity and to address any questions or doubts, a trained clinician was present to assist parents/caregivers during questionnaire completion. In order to avoid missing data, responses to every question were mandatory; therefore, all respondents were included in this study.

Since P-CPQ-16 and FIS-8 were not available in Italian, translation and cross-cultural adaptation processes were first performed, in accordance with established guidelines [14]. Three dentists who were fluent in both Italian and English, and knowledgeable about QoL terminology, translated P-CPQ-16 and FIS-8 from English to Italian. Afterwards, back translation was carried out by a doctor whose mother tongue was English and who was unaware of the terminology and the purpose of the study. A committee of three dentists, who were skilled in the English language and knowledgeable about QoL, compared original and back-translated versions of the questionnaires, reviewed the Italian version, and suggested modifications to improve semantic equivalence. In addition, a committee of two QoL and oral health experts evaluated the importance and meaning of items in the Italian versions and compared them to the original English version. Following this process, a sample of 21 parents and caregivers who were excluded from the final sample were used to pre-test the initial iterations of the Italian P-CPQ-16 and FIS-8. The objective of the pilot study was to discuss item suitability and to identify any comprehension difficulties. After being briefed on the pre-test’s objectives, participants were asked to rate each question’s overall comprehensibility and to re-report any that they found challenging to understand. During the pilot study, there were no misunderstandings of the translated items that were constantly repeated among all participants. Therefore, the first drafted version of the translated questionnaires was deemed suitable for more extensive use. Responses were graded on a 5-point Likert scale ranging from “never” (0) to “everyday/almost every day” (4) for the P-CPQ-16 and FIS-8 items, which assessed the frequency of the reported incidents during the last three months [11,13]. Additionally, there was a “Don’t know” option that yielded zero points. The sum of the individual item scores yields the overall score and domain scores. Higher scores indicate a greater negative impact on the patient’s QoL and a greater negative impact of children’s oral condition on the family’s quality of life, based on the parents’/caregivers’ perception.

The P-CPQ-16 and FIS-8 were administered along with two standardized instruments: the Decayed Missing Filled Teeth (DMFT/dmft) index and Dental Aesthetic Index (DAI). The DMFT/dmft index (number of decayed, missing due to caries, and filled teeth in the permanent/primary dentition) is used to assess the severity or frequency of caries on individuals [15], and a high score suggests poor oral hygiene and progression of dental caries [16].

Ten occlusal characteristics—overjet, mandibular overjet, tooth loss, diastema, anterior open bite, anterior diastema, anterior crowding, the largest maxillary anterior irregularities, the largest mandibular anterior irregularities, and sagittal molar relationship—are included in the DAI, which is used to evaluate the degree of malocclusion in the aesthetic region [17]. A DAI score of 0–25 means that there is little-to-no need for treatment; a score of 26–30 means that therapy is elective; a score of 31–35 means that treatment is extremely desirable; and a score of 36 or more means that treatment is mandatory.

Based on data available in the literature [18], a total of 128 patients are required to identify differences among the means of the four groups using a one-way ANOVA and considering an alpha error of 0.05, a power of 80%, and an effect size of f = 0.3. Data distribution was evaluated using the Shapiro–Wilk test. To evaluate if the severity of malocclusion or compromised oral health had an impact on the participants’ quality of life, both the Kruskal–Wallis test and regression analysis were performed. The scores of each P-CPQ-16 and FIS-8 domain were analyzed according to the groups of DMFT and DAI scores with the Kruskal–Wallis test and according to the continuous scores of the DMFT and DAI by regression analysis. For the first part, the participants were divided into 3 groups according to the DMFT/dmft scores: Group 1—DMFT + dmft = 0, Group 2—1 ≤ DMFT + dmft ≤ 3, Group 3—DMFT + dmft > 3. According to the DAI score, they were divided into 4 groups: Group 1—DAI < 25, Group 2—26 < DAI < 30, Group 3—31 < DAI < 35, Group 4—36 < DAI. A significance threshold of *p* < 0.05 was established. Spearman correlation (r) between P-CPQ-16, FIS-8, DMFT, and DAI was also used to analyze the data.

Furthermore, the structure of the P-CPQ-16 and FIS-8 were assessed using explanatory and confirmatory factor analysis. Lastly, Cronbach’s alpha coefficient (ɑ) for the subscales was used to determine internal consistency. A two-sided 95% confidence interval with a width of 0.16 when ICC = 0.81 and a two-sided 95% confidence interval with a width of 0.089 when ICC = 0.90 were produced by a sample of 41 individuals with two observations each, with *p* < 0.05.

## 3. Results

The P-CPQ-16 and FIS-8 were administered on a sample of 160 participants, of whom 68.8% were the patients’ mothers. The majority of the adolescent patients belonged to Group 1 of the DAI (55.6%) and DMFT (49.3%), which indicated no or slight treatment need and good oral hygiene, as well as low progression of dental caries, respectively.

Mean and standard deviation values of each domain of P-CPQ-16 in the current population were as follows: oro-functional alterations: 6.3 ± 4.2; social well-being: 2.8 ± 2.6; emotional well-being: 3.7 ± 3.1; and eating disturbances: 1.4 ± 1.5, while the mean and standard deviation values of the domain of FIS-8 were 7.4 ± 5.6.

Scores of total and all domains of P-CPQ-16 did not show any significant difference in relation to the DAI (Table 1) and the DMFT/dmft (Table 2) subgroups.

Similarly, FIS-8 scores did not show any significant difference among the subgroups of the DAI (Table 3) and the DMFT/dmft (Table 4) scores.

The regression analysis revealed a statistically significant association between the DAI (Table 1) and DMFT (Table 2) scores with respect to the total score and the social well-being domain of the P-CPQ-16.

The Spearman correlation did not show any statistically significant correlation between P-CPQ-16 and FIS-8 domains in relation to the DAI and DMFT scores, except for the social well-being domain of P-CPQ-16 with respect to DAI scores (Table 1).

The explanatory factor analysis for FIS-8 confirmed the structure with one factor as in the original English version; for the P-CPQ-16, the explanatory analysis detected four factors as the original P-CPQ-16 but with different distribution of items for each domain. The distribution of items for domains in the original and in the translated Italian version are reported in the Supplementary Materials.

The confirmatory analysis was performed with the original structure of P-CPQ-16 (comparative fit index = 0.857) and was then repeated with the new structure of P-CPQ-16 and FIS-8, showing that the new forms had better model fit indices (comparative fit index = 0.867 I-P-CPQ-16, comparative fit index = 0.865 I-FIS-8); therefore, the authors decided to use the new domains.

The Cronbach’s alpha coefficient (ɑ) of the PCPQ-16 domains ranged between 0.54 (eating disturbances domain) and 0.77 (oro-functional alterations domain) (Table 5), while the ICC FIS-8 measured 0.86.

Test–retest reliability (ICC) of the P-CPQ-16 ranged between 0.63 (emotional well-being domain) and 0.94 (oro-functional alterations domain) (Table 5), while for the FIS-8 it ranged between 0.58 and 0.81 with 0% of missing items.

## 4. Discussion

The aim of this study was to measure parental/caregivers’ perceptions of the impact of children’s oral-health-related quality of life (OHRQoL), based on the severity of their children’s malocclusion in the aesthetic region and their dental caries status, as well as assess the impact of children’s orofacial conditions on the family.

The social well-being domain and total score of the P-CPQ-16 showed significantly different scores when divided according to malocclusion severity (measured with the DAI) and the severity of caries (measured with the DMFT/dmft), which shows the P-CPQ-16’s capacity to differentiate between various degrees of malocclusion and caries. Similar results were found in the literature, where authors pointed out significant differences between the total scores of P-CPQ and malocclusion severity [19,20]. Furthermore, Albites et al. [21] found a significant difference in the total scores of the P-CPQ compared to caries severity, while Kumar et al. [22] did not find any significant differences between P-CPQ-16 and DMFT/dmft scores. Notably, the impact of dental conditions on social well-being, especially those affecting visible areas, was found to be closely related to social interactions [23]. This supports our finding that the effects of caries severity and malocclusion of adolescents on their social well-being-related OHRQoL may play a significant role from parental perceptions. These results are further supported by a study by Abreu et al., where parents reported improved social well-being related to OHRQoL during fixed appliance therapy for their adolescent children [24].

On the other hand, the remaining three domains of P-CPQ-16 did not show any significance among the subgroups of DAI and DMFT/dmft scores. In a study by Goursand et al., even the mean total scale score of P-CPQ was higher in the malocclusion group; it did not achieve a statistically significant difference between the subgroups, which was explained by the fact that the DAI only evaluates aesthetic characteristics of malocclusion, while the impact of the malocclusion might also be due to factors located in the posterior area (such as a posterior cross bite), that are not evaluated by the DAI [25]. It is crucial to note that parental perceptions of caries and malocclusion may frequently diverge from the objective severity determined by clinical indicators [26]. This diversion may occur because parents’ impressions are shaped by their social background and personal experiences, which may not always coincide with the clinical severity of the caries or malocclusion.

The subgroups of DAI and DMFT/dmft scores for the FIS-8 failed to show any statistically significant differences, indicating that the quality of life of Italian families is not significantly impacted by the severity of malocclusion and caries in their children. Controversial findings have been found in the literature, with some studies being in line with the current findings [22,27], in addition to additional research showing that dental caries significantly impact family life [19]. However, severe dental caries were recognized as the main risk factor in several of these studies where a correlation was found (number of teeth with pulp engagement, ulceration, fistula, or abscess), which might have a greater impact on families. In the early stages, dental caries may not have an impact on a child’s capacity to carry out everyday activities with their family, despite the fact that it is relatively common and is expected in low-income populations [28]. In accordance with our results, Piassi et al. found that the total scale and subscales scores of FIS varied according to the categories of malocclusion severity, but they did not find any significant statistical differences among them [27]. However, Pipovic et al. showed that malocclusions had a significant effect on a child’s family [29].

According to Locker and Slade, the kind or origin of the disease, socio-demographic characteristics, and variance in individuals’ perception levels may all be contributing factors to the limited relationship observed between parental perception/family impact and the clinical variables under investigation [30]. Still, it was interesting to note that although the current study has been performed on a patient sample, meaning people attending the university clinic for a first consultation and seeking dental treatment for their children, the impact of dental problems of their children (both malocclusion and dental caries) on the family burden was quite low. This can be explained by the fact that the patients did not have any previous dental treatment, so their parents/caregivers did not take days off from work to bring their children to appointments or become economically affected.

The first drafted versions of the P-CPQ-16 and FIS-8 did not require major changes during cross-cultural adaptation and the pre-test, most likely because of an affinity between Canadian and Italian socio-cultural environments where the questionnaires were developed and assessed. Conversely, in a Telugu-speaking population of India, multiple adjustments were required before creating a final cross-culturally adapted version of the FIS-8 and P-CPQ-16 [22]. The factorial analysis for the I-P-CPQ-16 confirmed a four-domain structure, like the original one; however, for the I-FIS-8, it confirmed a one-domain structure, different from the original version.

According to the interpretation of George et al. [31], the ɑ of the I-PCP-Q-16 and I-FIS-8 showed between poor and good internal consistency, similar to the Peruvian Spanish validation of PCP-Q [21] and FIS [32], where the internal consistencies were found to be between poor to acceptable [21]. Poor values were observed only for the “eating disturbances” domain of I-PCP-Q-16, and this may be the result of the subscale having a small number of items (two items). As a matter of fact, data indicate that reliability estimates rise with scale length [33]. Another reason for the poor scoring can be due to the fact that items included in this subscale refer to “food caught in or between the teeth” and “food stuck in the roof of the mouth”, which might be of particular interest when an orthodontic device is present in the mouth. Since data for the current study were collected before any kind of orthodontic treatment, the questions may not have been perceived as important as a result.

Regarding ICC reliability, except for one item of I-PCP-Q-16 with poor reliability, all the items of I-PCP-Q-16 and I-FIS-8 had between moderate and excellent reliability [34]. Similarly, Dimberg et al. reported test–retest reliability for the Swedish validation of the PCP-Q as ranging from poor to excellent [35]. The percentage of missing items was all 0% because all items required a mandatory response. The high variability of internal consistency in the study could be due to the high heterogeneity of the population that was looking for a first consultation at the university hospital.

A limitation of our study was that it was not possible to analyze the socio-economic status of the parents/caregivers with respect to the scores of the questionnaires. Additionally, our sample consisted of a treatment-seeking population, which may not represent the general population. Future studies should include socio-economic status in the analysis and assess whether parental perception and family impact change after multiple dental treatments or a period of orthodontic care.

## 5. Conclusions

Parents/caregivers perceived a difference in their children’s OHRQoL according to different severities of malocclusion and dental caries; however, they did not report any impact on their family’s quality of life, which may be attributed to the influence of other confounding factors, such as social, environmental, and personal features. It is likely that, before starting any dental treatment, parents/caregivers’ quality of life could not be strongly impacted by their children’s OHRQoL.

Since adolescents often visit the dentist due to parental influence, and because the parents of children with severe malocclusions or compromised oral health perceive a lower quality of life for them, addressing parents’ concerns and involving them in treatment decisions is crucial for more effective care.

## Figures and Tables

**Table 1:** Results of Kruskal–Wallis, Spearman’s correlation, and regression analysis of the P-CPQ-16 scores according to different scores of the DAI.

	Scores of P-CPQ-16 (Mean ± SD)				
DAI	Oro-Functional Alterations	Social Well-Being	Emotional Well-Being	Eating Disturbances	TOTAL
Group 1 (*n* = 89)	6.1 ± 4.22	2.3 ± 2.3	3.5 ± 2.8	1.46 ± 1.53	13.47 ± 8.24
Group 2 (*n* = 23)	5.3 ± 2.5	3.4 ± 2.9	3.8 ± 3.0	1.3 ± 1.1	13.81 ± 5.48
Group 3 (*n* = 18)	7.6 ± 4.7	3.4 ± 2.3	5.0 ± 3.6	1.6 ± 2.1	17.7 ± 10.2
Group 4 (*n* = 30)	7.0 ± 4.8	3.4 ± 3.0	3.5 ± 3.5	1.63 ± 1.42	15.56 ± 9.4
*p*	*p* = 0.377	*p* = 0.877	*p* = 0.104	*p* = 0.436	*p* = 0.341
Spearman’s correlation	r = 0.089	r = 0.191	r = 0.035	r = 0.054	r = 0.141
	*p* = 0.264	*p* = 0.015 *	*p* = 0.662	*p* = 0.495	*p* = 0.075
Regression	β = 0.052	β = 0.049	β = 0.019	β = 0.013	β = 0.133
	*p* = 0.065	*p* = 0.005 *	*p* = 0.370	*p* = 0.215	*p* = 0.019 *

*p* = *p* value, r = correlation coefficient of the Spearman correlation, β = association coefficient of the regression analysis, * = *p* < 0.05. Group 1—DAI < 25, Group 2—26 < DAI < 30, Group 3—31 < DAI < 35, Group 4—36 < DAI.

**Table 2:** Results of Kruskal–Wallis, Spearman’s correlation, and regression analysis of the PCPQ-16 scores according to different scores of the DMFT/dmft index.

	Scores of P-CPQ-16 (Mean ± SD)				
DMFT + dmft	Oro-Functional Alterations	Social Well-Being	Emotional Well-Being	Eating Disturbances	TOTAL
Group 1 (*n* = 79)	6.04 ± 4.2	2.5 ± 2.3	3.64 ± 3.0	1.35 ± 1.41	13.62 ± 8.2
Group 2 (*n* = 46)	6.13 ± 4.0	2.47 ± 2.44	4.13 ± 3.25	1.65 ± 1.7	14.39 ± 8.6
Group 3 (*n* = 35)	7.37 ± 4.4	3.77 ± 3.13	3.43 ± 3.2	1.47 ± 1.5	16.17 ± 8.66
*p*	*p* = 0.309	*p* = 0.636	*p* = 0.122	*p* = 0.536	*p* = 0.290
Spearman’s correlation	r = 0.127	r = 0.121	r = 0.001	r = 0.082	r = 0.142
	*p* = 0.109	*p* = 0.127	*p* = 0.989	*p* = 0.302	*p* = 0.073
Regression	β = 0.207	β = 0.185	β = 0.021	β = 0.039	β = 0.451
	*p* = 0.062	*p* = 0.007 *	*p* = 0.803	*p* = 0.335	*p* = 0.041 *

*p* = *p* value, r = correlation coefficient of the Spearman correlation, β = association coefficient of the regression analysis, * = *p* < 0.05. Group 1—DMFT + dmft = 0, Group 2—1 ≤ DMFT + dmft ≤ 3, Group 3—DMFT + dmft > 3.

**Table 3:** Results of Kruskal–Wallis, Spearman’s correlation, and regression analysis of the FIS-8 scores according to different scores of the DAI.

	Scores of FIS-8 (Mean ± SD)
DAI	TOTAL
Group 1 (*n* = 89)	7.1 ± 5.7
Group 2 (*n* = 23)	6.3 ± 4.7
Group 3 (*n* = 18)	9.1 ± 6.7
Group 4 (*n* = 30)	7.96 ± 4.9
*p*	*p* = 0.399
Spearman’s correlation	r = 0.081
	*p* = 0.311
Regression	β = 0.047
	*p* = 0.206

*p* = *p* value, r = correlation coefficient of the Spearman correlation, β = association coefficient of the regression analysis. Group 1—DAI < 25, Group 2—26 < DAI < 30, Group 3—31 < DAI < 35, Group 4—36 < DAI.

**Table 4:** Results of Kruskal–Wallis, Spearman’s correlation, and regression analysis of the FIS-8 scores according to different scores of DMFT/dmft index.

	Scores of FIS-8 (Mean ± SD)
DMFT + dmft	TOTAL
Group 1 (*n* = 79)	7.3 ± 5.4
Group 2 (*n* = 46)	6.7 ± 5.8
Group 3 (*n* = 35)	8.2 ± 5.5
*p*	*p* = 0.365
Spearman’s correlation	r = 0.055
	*p* = 0.492
Regression	β = 0.126
	*p* = 0.391

*p* = *p* value, r = correlation coefficient of the Spearman correlation, β = association coefficient of the regression analysis. Group 1—DMFT + dmft = 0, Group 2—1 ≤ DMFT + dmft ≤ 3, Group 3—DMFT + dmft > 3.

**Table 5:** Internal consistency for the PCPQ-16 subscales (N = 160) and test–retest reliability assessed by intraclass correlation coefficient (N = 41).

	α	ICC	MI
Oro-Functional Alterations	0.77	0.712–0.941	0%
Social Well-Being	0.67	0.470–0.868	0%
Emotional Well-Being	0.76	0.637–0.737	0%
Eating Disturbances	0.54	0.787–0.904	0%

ICC: Intraclass correlation coefficient for each item of the domain; α: Cronbach’s alpha; MI: percentage of missing items.

## Data Availability

The datasets used and analyzed during the current study are available from the corresponding author on reasonable request.

## Supplementary Materials

The following supporting information can be downloaded at https://www.mdpi.com/article/10.3390/children12040425/s1, Table S1: Distribution of items for domains in the original and in the translated Italian version of PCP-Q; Table S2: Distribution of items for domains in the original and in the translated Italian version of FIS.
