# Supplementary material for: Parental Perceptions and Family Impact on Adolescents’ Oral Health-Related Quality of Life in Relation to the Severity of Malocclusion and Caries Status
Source: Children (Basel). 2025 Mar 28;12(4):425. doi: 10.3390/children12040425 (PMC12025449; doi:10.3390/children12040425)
Supplement: Supplementary file 1 [file children-12-00425-s001.zip › children-3498977-supplementary.pdf]

**SUPPLEMENTARY MATERIAL**

**Table S1: Distribution of items for domains in the original and in the translated Italian version of PCP-Q**

| During the last 3 months, how often has your child (had/been)… | | |
|---|---|---|
| | **New Domain** | **Original Domain** |
| **Pain in the teeth, lips, jaws or mouth** | Oro-Functional Alterations | OS |
| **Bad breath** | Oro-Functional Alterations | OS |
| **Taken longer than others to eat a meal** | Oro-Functional Alterations | FL |
| **Difficulty biting or chewing firm foods** | Oro-Functional Alterations | FL |
| **Had trouble sleeping** | Oro-Functional Alterations | FL |
| **Breathed through the mouth** | Oro-Functional Alterations | FL |
| **Missed school or preschool** | Social Well-Being | SW |
| **Not wanted to talk to other children** | Social Well-Being | SW |
| **Had a hard time paying attention in school** | Social Well-Being | SW |
| **Avoided smiling or laughing when around other children** | Social Well-Being | SW |
| **Been upset** | Emotional Well-Being | EW |
| **Been irritable or frustrated** | Emotional Well-Being | EW |
| **Been anxious or fearful** | Emotional Well-Being | EW |
| **Acted shy or embarrassed** | Emotional Well-Being | EW |
| **Food caught in or between the teeth** | Eating Disturbances | OS |
| **Food stuck in the roof of the mouth** | Eating Disturbances | OS |

**Table S2: Distribution of items for domains in the original and in the translated Italian version of FIS**

| During the last 3 months, | | |
|---|---|---|
| | **FIS** | **Original Domain** |
| **Have you or has the other parent taken time off work?** | 1 | PA |
| **Has your child required more attention from you or the other parent?** | 1 | PA |
| **Have you or has the other parent had less time for yourself or other family members?** | 1 | PA |
| **Has your sleep or that of the other parent been disrupted?** | 1 | PA |
| **Have you or has the other parent been upset?** | 1 | PE |
| **Have you or has the other parent felt guilty?** | 1 | PE |
| **Has your child argued with you or the other parent?** | 1 | FC |
| **Has your child blamed you or the other parent?** | 1 | FC |
